# Evaluation of the fetal abdomen by magnetic resonance imaging. Part 1: malformations of the abdominal cavity

**DOI:** 10.1590/0100-3984.2016.0140

**Published:** 2018

**Authors:** Ana Paula Pinho Matos, Luciana de Barros Duarte, Pedro Teixeira Castro, Pedro Daltro, Heron Werner Júnior, Edward Araujo Júnior

**Affiliations:** 1 MD, Specialist in Fetal Medicine, Masters Student, Department of Maternal and Child Care, Universidade Federal Fluminense (UFF), Niterói, RJ, Brazil.; 2 PhD, Adjunct Professor, Department of Maternal and Child Care, Universidade Federal Fluminense (UFF), Niterói, RJ, Brazil.; 3 MSc, MD, Department of Radiology, Clínica de Diagnóstico por Imagem (CDPI), Rio de Janeiro, RJ, Brazil.; 4 PhD, MD, Department of Radiology, Clínica de Diagnóstico por Imagem (CDPI), Rio de Janeiro, RJ, Brazil.; 5 Tenured Adjunct Professor, Department of Obstetrics, Escola Paulista de Medicina da Universidade Federal de São Paulo (EPM-Unifesp), São Paulo, SP, Brazil.

**Keywords:** Fetus, Congenital abnormalities/diagnostic imaging, Abdomen/diagnostic imaging, Magnetic resonance imaging, Feto, Anormalidades congênitas/diagnóstico por imagem, Abdome/diagnóstico por imagem, Ressonância magnética

## Abstract

Although ultrasound continues to be the mainstay modality for the evaluation of fetal disorders, fetal magnetic resonance imaging (MRI) has often been used as a valuable adjunct in recent years. The exponential growth of the use of fetal MRI has been facilitated by technological advancements such as ultrafast T2-weighted sequences and diffusion-weighted imaging. Fetal MRI can achieve results that are comparable to or better than those of ultrasound, particularly in cases of maternal obesity, severe oligohydramnios, or abnormal fetal position. Because of its superior soft tissue contrast, wide field of view, and multiplanar imaging, fetal MRI is able to evaluate the large fetal organs, such as the lungs, liver, bowel, and kidneys. In addition, fetal MRI allows large or complex malformations to be examined, facilitating the understanding of the malformation within the context of the body as a whole. Initial fetal MRI studies were focused on the central nervous system. With advances in software and hardware, fetal MRI gained importance in the evaluation of the fetal abdomen. The purpose of this article is to review the recent literature and developments in MRI evaluation of the fetal abdomen, with an emphasis on imaging aspects, protocols, and common clinical indications.

## Abstract (pt)

Apesar de a ultrassonografia (US) permanecer como principal método na avaliação de desordens fetais, a ressonância magnética (RM) fetal tem sido frequentemente usada como método adjuvante nos últimos anos. O crescente uso da RM fetal foi facilitado pelos avanços tecnológicos como a sequência pesada em T2 ultrarrápida e imagens *diffusion-weighted*. A RM fetal pode alcançar resultados superiores ou semelhantes aos da US, principalmente em casos de obesidade materna, oligo-hidrâmnio ou posição fetal anômala. Por apresentar melhor contraste entre tecidos, grande campo de visão e cortes multiplanares, a RM fetal é capaz de avaliar órgãos fetais de grande volume como pulmões, fígado, cólon e rins. Ademais, a RM fetal permite o exame de malformações grandes ou complexas, facilitando a compreensão da malformação no contexto de todo o corpo fetal. Inicialmente, os estudos eram direcionados ao sistema nervoso central. Com o avanço dos *softwares* e *hardwares*, a RM fetal ganhou importância na avaliação da cavidade do abdome fetal. O propósito deste artigo é revisar a literatura recente e avanços na avaliação da cavidade abdominal fetal pela RM, com ênfase nas características das imagens, protocolos e indicações clínicas mais comuns.

## INTRODUCTION

The importance of imaging methods in the diagnosis of congenital malformations^(1-3)^, especially in fetal medicine^(4-7)^, has been the objective of a series of recent studies conducted in Brazil. Because of improvements in image resolution, together with the development of tissue-specific contrast agents, increases in the speed of image acquisition, and the availability of software for image processing, as well as its wide field of view, magnetic resonance imaging (MRI) has become an important tool in fetal diagnostics. Ultrasound continues to be the preferred method of screening for fetal anomalies^(8)^, because of its low cost and ready availability. However, certain conditions, such as oligohydramnios, maternal obesity, and unfavorable fetal position, reduce the efficiency of ultrasound to make an accurate prenatal diagnosis and call for the use of fetal MRI. Due to the difficulty in characterizing malformations of the fetal abdominal cavity, fetal MRI assessment can be necessary, to add prognostic information or to aid in therapeutic planning, when the ultrasound findings are inconclusive^(9)^.

## MALFORMATIONS OF THE ABDOMINAL CAVITY

### Esophageal atresia

**Figure 1:**
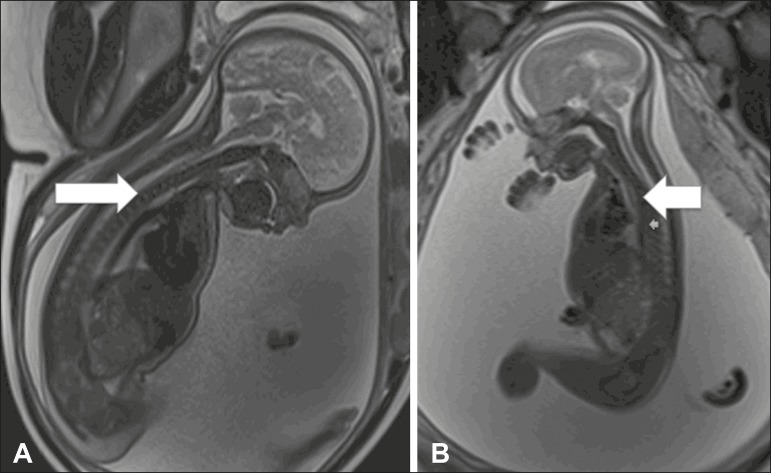
Esophageal atresia. **A:** Fetus at 30 weeks of gestation showing polyhydramnios and dilation of the esophagus (arrow). **B:** Fetus at 22 weeks of gestation showing polyhydramnios and dilation of the esophagus (arrow).

Esophageal atresia originates from malformation of the tracheoesophageal septum before 8 weeks of gestation. With an incidence ranging from 1/2500 to 1/4000 live births, the prognosis of esophageal atresia is dependent on the presence of associated malformations. It can present as an isolated malformation, a less common form, or can be accompanied by tracheoesophageal fistula, a more common form that is seen in 90% of cases^(10)^. Although esophageal atresia is technically a thoracic malformation, we report it here because it relates to digestive malformations. The strong association with fistula results in the underdiagnosis of esophageal atresia during prenatal screening. A fistula can divert amniotic fluid to the stomach, making it difficult to detect anomalies of the digestive tract because the most characteristic signs of such anomalies, including an empty stomach and polyhydramnios, are absent. In some cases, the most proximal portion of the atresia can be filled with amniotic fluid (Figure 1).

Esophageal atresia is suspected when the stomach is not visible or is smaller than expected, as well as when such features are accompanied by polyhydramnios. Although ultrasound evaluation of the esophagus provides information regarding its anatomy and motility, it is an inefficient means of acquiring images of the cervical segment and gastroesophageal junction. In esophageal atresia, ultrasound shows the esophagus as two echogenic lines corresponding to the anterior and posterior walls. In 90% of cases, the upper sphincter can be seen to open during the swallowing of fluid, usually after 19 weeks of gestation, although esophageal maturation is considered complete only after 32 weeks.

In T2-weighted MRI sequences, the esophagus presents a signal that is isointense or hypointense, with an occasional hyperintense (amniotic fluid) signal. It is also possible to determine esophageal mobility by using MRI to evaluate swallowing of the amniotic fluid. On T2-weighted sequences, sagittal slices can show the amniotic fluid, appearing as a hyperintense signal, moving through the oral cavity and toward the stomach. MRI facilitates the assessment of the cervical segments and gastroesophageal junction. In esophageal atresia, dilation of the proximal esophagus and hypopharynx is not uncommon. Fluid collected in the pouch at the bottom of the malformation is more easily seen by MRI.

### Duodenal obstruction

The most common type of intestinal atresia, occurring in 1 out of every 5000 live births^(11)^, is duodenal obstruction, which results from the persistence of luminal obliteration at 8 to 10 weeks of gestation. Duodenal obstruction can also be secondary to extrinsic compression by the portal vein or upper mesenteric artery, with a clinical profile similar to that of luminal obliteration. Trisomy 21 and congenital heart disease occur in one third of all cases of duodenal obstruction^(12)^. Ultrasound examination of duodenal obstruction shows hyperperistalsis, with the classic double-bubble sign, although fetal regurgitation can temporarily eliminate the double-bubble image. Its diagnosis before the second trimester is rare due to the immaturity of the gastrointestinal system. Early diagnosis of duodenal obstruction is associated with other malformations.

**Figure 2:**
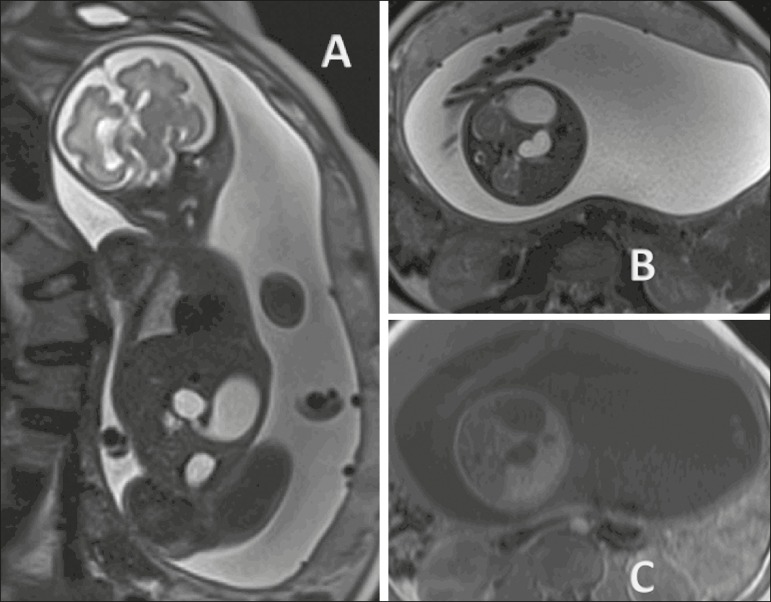
Duodenal atresia in a fetus at 32 weeks of gestation. **A:** Coronal T2-weighted sequence showing a dilated stomach, pylorus, and duodenal dilation. **B:** Axial T2-weighted sequence. **C:** Axial T1-weighted sequence showing the stomach and duodenal dilation.

In cases of duodenal obstruction, MRI reveals dilation of the stomach and duodenum, both of which show a hyperintense signal, due to the presence of amniotic fluid, in T2-weighted sequences. The distal intestine should be evaluated in order to differentiate between incomplete duodenal obstruction and duodenal atresia (Figure 2). In cases of incomplete obstruction, meconium fills the jejunum and colon. The contents of the distal intestinal can show a meconium-like signal, albeit with lower signal intensity in T1-weighted sequences and a decrease in intestinal diameter^(13)^. In duodenal atresia/obstruction, MRI adds valuable information for the diagnostic investigation of this malformation in the presence of stenosis or diaphragm in the pylorus, because there is a hypointense signal in T1-weighted sequences of the distal intestine. MRI is also more accurate in the detection of extrinsic obstructive masses, such as annular pancreas.

## PERFUSION IN THE ABDOMINAL CAVITY

### Meconium peritonitis

Meconium peritonitis occurs in 1 out of every 2000 live births, which makes it the most common complication of fetal intestinal occlusion. It is characterized as an inflammatory response due to the chemical reaction of the peritoneum to the presence of meconium. In the absence of prenatal diagnosis and planned postnatal treatment, the perinatal mortality associated with meconium peritonitis is reported to be as high as 62%^(14)^. Ultrasound reveals effusion in the abdominal cavity. Meconium peritonitis differs from ascites, because in the meconium peritonitis there are hyperechoic areas (calcifications) in the abdomen or scrotum, together with intestinal dilation and polyhydramnios^(15)^.

**Figure 3:**
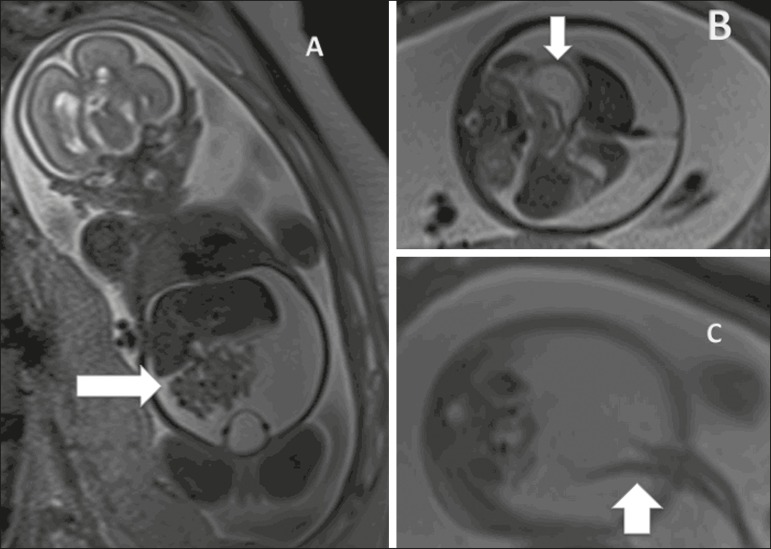
Idiopathic ascites in a fetus at 28 weeks of gestation. **A:** Distended abdomen with a fluid collection showing a hyperintense signal in a coronal T2-weighted sequence. Well-defined intestinal loops (arrow). **B:** Axial image of the fetal abdomen showing ascites. Note the organs floating in the abdominal cavity. Stomach (arrow). **C:** Axial image of the fetal abdomen showing ascites. Umbilical arteries (arrow) individualized by the large volume of liquid in the abdominal cavity.

In the presence of intestinal dilation and effusion in the abdominal cavity, MRI is an important diagnostic tool in the differential diagnosis between ascites and meconium peritonitis. In T1-weighted sequences, meconium peritonitis shows a heterogeneous signal that is intermediate in comparison with that of the amniotic fluid, whereas the signal is hyperintense and heterogeneous in T2-weighted sequences. Meconium peritonitis can also present as a large pseudocyst, with the same characteristics previously described (Figure 3).

## ABDOMINAL CYSTS

### Ovarian cysts

**Figure 4:**
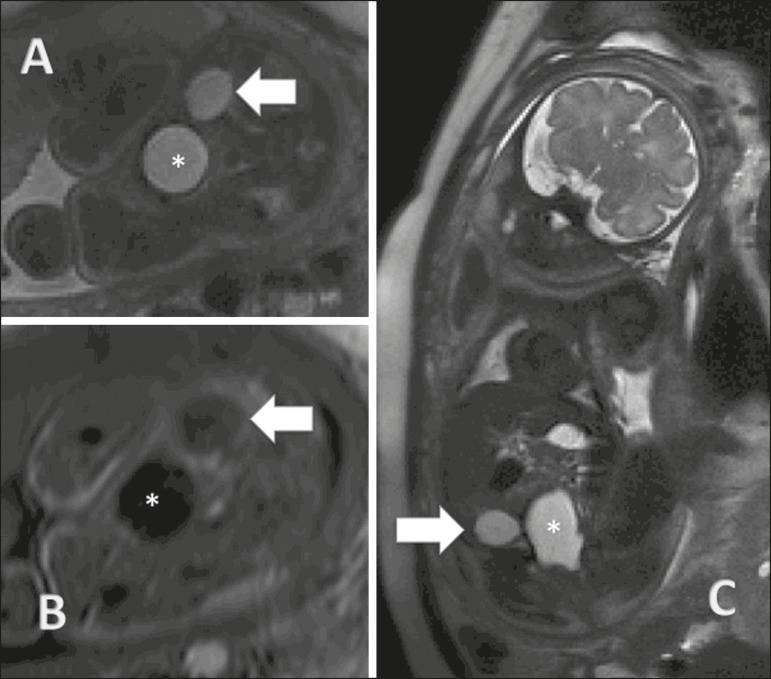
Ovarian cyst in a fetus at 32 weeks of gestation. **A:** Axial T2-weighted sequence of the fetal pelvis showing a hyperintense signal in the right adnexal region. **B:** Axial T1-weighted sequence of the fetal pelvis, showing a well-defined area of signal hypointensity, with homogeneous contours, in the right adnexal region. **C:** Coronal T2-weighted sequence showing a cyst with a hyperintense signal in the right adnexal region. Ovarian cyst (arrows) and fetal bladder (asterisks).

In female fetuses, ovarian cysts are the main causes of abdominal masses. The reported incidence of neonatal ovarian cysts is over 30%. Ovarian cysts are only rarely accompanied by other malformations and resolve spontaneously in most cases. In the neonatal period, the most common complications associated with ovarian cysts are ovarian torsion, hemorrhage, and rupture of the cyst, any of which can require surgery. In addition to expectant management, therapeutic options include needle aspiration and laparotomy. The typical presentation is that of a simple cyst. In heterogeneous masses, intracystic hemorrhage and ovarian torsion should be suspected. In practical terms, a diagnosis of ovarian cyst should be considered when a female fetus presents with a pelvic cyst in the absence of urinary or gastrointestinal malformations. Ovarian cysts typically occur in the flanks and iliac fossae (Figure 4). When the cyst is central, a diagnosis of mesenteric cyst should be considered^(16)^.

### Mesenteric cysts

**Figure 5:**
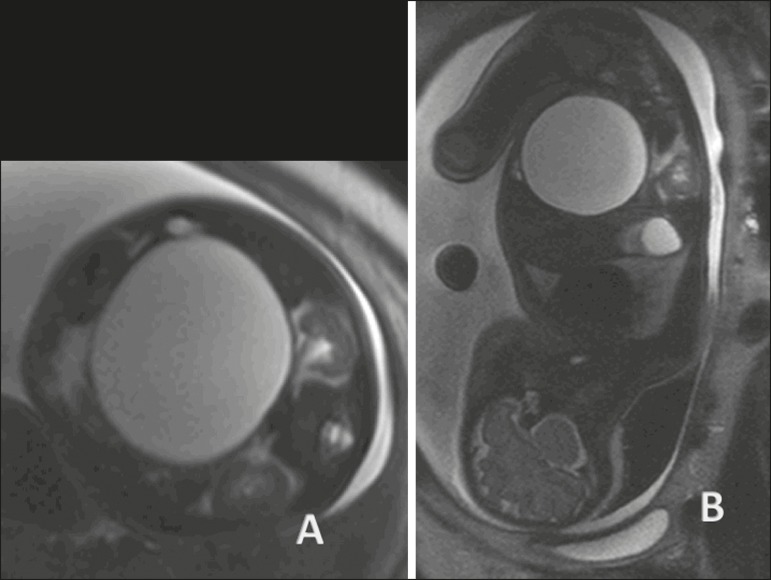
Mesenteric cyst in a fetus at 32 weeks of gestation. Abdominal mass with homogeneous content, a thin capsule, and regular contours, centralized in the abdomen and showing a hyperintense signal in a T2-weighted sequence.

Although mesenteric cysts can arise as early as the first trimester, they are usually diagnosed after the second trimester. They are thin-walled cysts, without peristalsis, varying in size, and containing liquid. Mesenteric cysts are retroperitoneal (Figure 5) and are separated from the colon. They are related to lymphatic malformations and should be included in the differential diagnosis of renal anomalies, especially duplications.

Mesenteric cysts are described, mainly in children, as abdominal masses that are usually asymptomatic but can provoke gastrointestinal clinical symptoms suggestive of obstruction. They usually present as an isolated malformation, and the standard treatment is surgical, although it is possible to use sclerosing agents such as bleomycin.

## HEPATIC AND SPLENIC ANOMALIES

The most common hepatic alterations occurring *in utero* are calcifications, which can originate from a tumor, an infection, or an ischemic insult. Although ultrasound is the most accurate method for the evaluation of focal hepatic lesions, MRI has emerged as an important method for the evaluation of liver tumors, aiding in the differential diagnosis of hepatoblastoma, hemangioma, and neuroblastoma, as well as for the evaluation of the extent of the disease and involvement of the adjacent parenchyma. In liver diseases that involve the entire organ, MRI is of major value. When a hypointense signal is seen in T1- and T2-weighted sequences, hemosiderosis, hemochromatosis, and infectious diseases should be considered ^(17)^.

**Figure 6:**
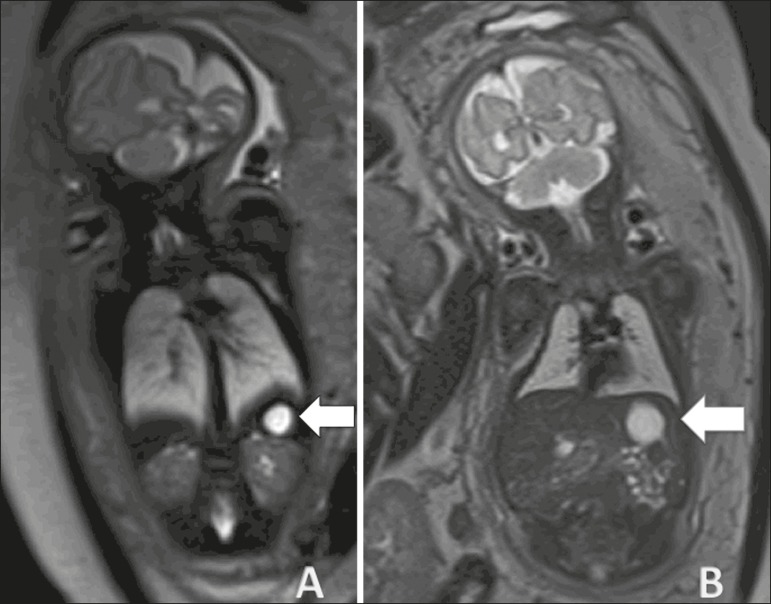
Splenic cyst. **A:** Coronal T2-weighted sequence of a fetus at 27 weeks of gestation showing a cyst with a hyperintense signal corresponding to a cyst with regular contours and thin, well-defined borders (arrow). **B:** Fetus at 29 weeks of gestation, showing a cyst with a hyperintense signal in a T2-weighted sequence, regular contours, and well-defined borders.

Splenic malformations can be visualized by MRI, which can also be used in order to confirm the diagnosis of splenic cysts, which are small (less than 2 cm in diameter) and hypointense, at the typical site within the spleen (Figure 6). The differential diagnosis includes neuroblastoma, and the prognosis of splenic cyst is good.
